# Bacterial Communities within Gingival Tissues from Periodontal Lesions Compared with Subgingival Plaque

**DOI:** 10.1080/20002297.2017.1325217

**Published:** 2017-06-01

**Authors:** Youngnim Choi

**Affiliations:** ^a^ Seoul National University, Seoul, South Korea

## Abstract

Periodontitis is caused by dysbiosis of subgingival plaque that results in increased bacterial invasion into gingival tissues. Although shifts in subgingival microbiota from healthy to periodontitis have been well characterized, the characteristics of bacterial communities located within gingival tissues have not been studied. To characterize microbiota within the tissues of periodontal lesions in comparison with plaque microbiota, gingival tissues and subgingival plaque were obtained from the same tooth of patients with periodontitis (n = 7). A pyrosequencing analysis of the 16S rRNA gene revealed that species richness and diversity were not significantly different between the two communities. However, inter-subject variation in intra-tissue communities was smaller than that in plaque communities. Intra-tissue communities were characterized by decreased Firmicutes and increased Fusobacteria, compared with the plaque communities. Particularly, *Fusobacterium nucleatum* and *Porphyromonas gingivalis* were highly increased within tissues, comprising 15–40% of the total bacteria. Furthermore, biofilm formation within the tissue was observed by Alcian Blue staining and atomic force microscopy, where degradation of fibers was prominent. Taken together, bacteria formed complex biofilm communities within gingival tissues that may serve as a reservoir for persistent infection. This novel finding may instigate new research into therapeutic strategies to treat periodontitis.

